# Acute medical care for individuals experiencing homelessness in Kuala Lumpur

**DOI:** 10.51866/mol.927

**Published:** 2025-06-10

**Authors:** Khasnur Abd Malek, Ilham Ameera Ismail, Farnaza Ariffin, Md Yasin Mazapuspavina, Raja Ahmad Shaharul

**Affiliations:** 1 MBChB, MFamMed, FRACGP, Primary Care Medicine Department, Faculty of Medicine, Universiti Teknologi MARA, Sungai Buloh Campus, Jalan Hospital, Sungai Buloh, Selangor, Malaysia. Email: drkhasnur@uitm.edu.my; 2 MBChB, Dr Fam Med, FAFP, FRACGP, Primary Care Medicine Department, Faculty of Medicine, Universiti Teknologi MARA, Sungai Buloh Campus, Jalan Hospital, Sungai Buloh, Selangor, Malaysia.; 3 MBBS, PMETB GP, FRCGP., Primary Care Medicine Department, Faculty of Medicine, Universiti Teknologi MARA, Sungai Buloh Campus, Jalan Hospital, Sungai Buloh, Selangor, Malaysia.; 4 MBBS, MMed Fam Med, Primary Care Medicine Department, Faculty of Medicine, Universiti Teknologi MARA, Sungai Buloh Campus, Jalan Hospital, Sungai Buloh, Selangor, Malaysia.; 5 MB BCh BAO, MRCGP, DGM, Pertubuhan Generasi Santuni Insan (Feeding Future Generation Society) (FFG) 9, Jalan 2, Taman Cempaka, Ampang, Kuala Lumpur, Malaysia.

**Keywords:** Homeless, Medical volunteers, Non-governmental organization, Corporate social responsibility

## Unmet health needs

Individuals experiencing homelessness often suffer from acute medical conditions that go untreated due to a lack of financial means to access healthcare services and delay in seeking medical help. To help address these challenges, the Food for Gelandangan (FFG) team, established in 2013, brings together community volunteers to distribute food and hygiene items every Friday night in the heart of Kuala Lumpur, along with healthcare professionals to provide medical care. However, volunteer doctors are limited, and medical services are only available sporadically depending on volunteer availability, with a family medicine specialist providing care once a month. In response, a dedicated group of family medicine specialists came together to develop a street-based acute medical care outreach programme to support the FFG’s initiative.

## The experience

**Figure 1:**
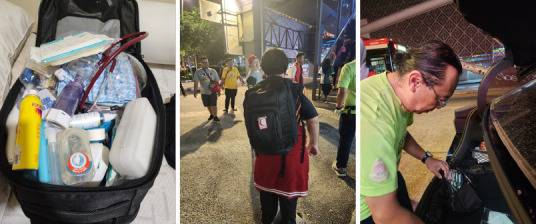
Medication bags filled with medicines and point-of-care assessment kits.

Our journey began with joining the Friday rounds to better understand the group’s needs, its logistical challenges and how to best provide acute medical care. Essential medications and medical equipment were purchased using individual funds to ensure adequate medical supply (Figure 1). Commonly needed medications include treatments for skin infections, fever, toothaches, mouth ulcers, pain, wounds, upper respiratory tract infections and chronic diseases. Additionally, there is a high demand for *minyak angin* (medicated oil).

**Figure 2:**
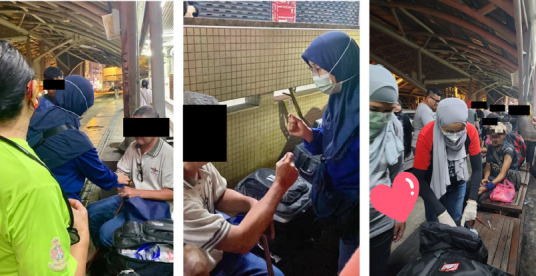
Volunteer family medicine doctors providing acute medical care to those in need.

**Figure 3:**
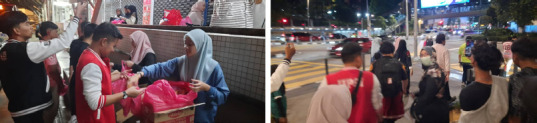
Volunteers packing non-perishable food and the group walk.

We arrived at the meet-up point, Kotaraya, by 9:30 pm. When we arrived, a long queue had already formed in an orderly manner, and our medical team began our service immediately, setting up a makeshift clinic on public benches (Figure 2). Other volunteers sorted donations including non-perishable food, hygiene items and mosquito coils (Figure 3).

**Figure 4:**
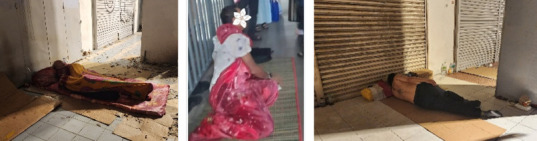
Unsheltered homeless and their so-called homes along the pedestrian pavement.

To truly reach individuals experiencing homelessness, we then walked the streets, carrying our medical bags through the city centre. It was heartbreaking to see individuals making sidewalks, bridges and dark alleys their homes. Many had no pillows or clean mattresses, just a thin piece of cardboard between them and the cold, dirty pavement. Streetlights illuminated their makeshift beds, and the constant noise of passing vehicles was their lullaby (Figure 4).

## Encountered medical issues

**Figure 5:**
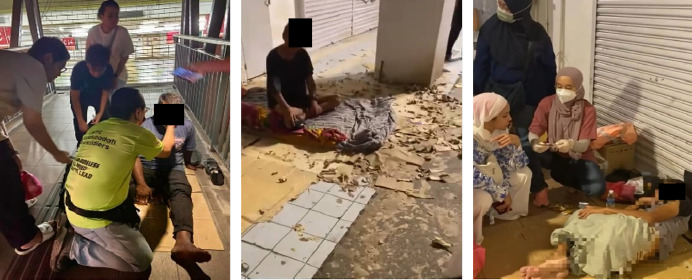
Providing aid to the homeless in harsh living conditions.

The medical issues of those approached by the team included skin infections, musculoskeletal pain and wounds, although some struggled with substance use and psychiatric illness. During our visits, we treated medical emergencies, including hypertensive urgency, asthma and pneumonia (Figure 5). Despite language barriers, we provided urgent care and even coordinated an ambulance service, ensuring a critically ill patient received hospital treatment and thus preventing severe complications.

## A solution for now

**Figure 6:**
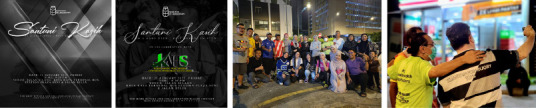
The Food for Gelandangan team and its amazing volunteers.

Street-based medical care reduces access barriers and builds trust. A consistent outreach programme (Figure 6) can improve health, reduce emergencies and encourage better health-seeking behaviours, ultimately lowering morbidity and mortality among homeless individuals.

## The future

Sustaining medical care requires volunteers, government support, NGO collaboration and CSR funding to impact vulnerable communities effectively. The dream is clear, a future where every individual, regardless of their circumstances, has access to essential acute healthcare. With collective effort and firm dedication, we can turn this vision into reality.

